# Alterations of miRNA Expression in Diffuse Hyperplastic Perilobar Nephroblastomatosis: Mapping the Way to Understanding Wilms’ Tumor Development and Differential Diagnosis

**DOI:** 10.3390/ijms24108793

**Published:** 2023-05-15

**Authors:** Ádám Csók, Tamás Micsik, Zsófia Magyar, Tamás Tornóczky, Levente Kuthi, Yumika Nishi, Krisztina Szirák, Monika Csóka, Gábor Ottóffy, Beáta Soltész, István Balogh, Gergely Buglyó

**Affiliations:** 1 Department of Human Genetics, Faculty of Medicine, University of Debrecen, 4032 Debrecen, Hungary; csok.adam@med.unideb.hu (Á.C.); buglyo.gergely@med.unideb.hu (G.B.); 2 Department of Pathology and Experimental Cancer Research, Semmelweis University, 1085 Budapest, Hungary; 3 Department of Obstetrics and Gynaecology, Baross Street Division, Semmelweis University, 1088 Budapest, Hungary; 4 Department of Pathology, University of Pécs Medical School and Clinical Center, 7624 Pécs, Hungary; 5 Department of Pathology, Faculty of Medicine, Albert Szent-Györgyi Medical School, University of Szeged, 6725 Szeged, Hungary; 6 Department of Paediatrics, Semmelweis University, 1094 Budapest, Hungary; 7 Department of Pediatrics, University of Pécs Medical School and Clinical Center, 7623 Pécs, Hungary; 8 Division of Clinical Genetics, Department of Laboratory Medicine, Faculty of Medicine, University of Debrecen, 4032 Debrecen, Hungary

**Keywords:** Wilms’ tumor, nephrogenic rest, diffuse hyperplastic perilobar nephroblastomatosis, miRNA, formalin-fixed, paraffin-embedded sample

## Abstract

Wilms’ tumor (WT) is the most common renal malignancy in children. In diffuse hyperplastic perilobar nephroblastomatosis (DHPLN), nephrogenic rests result in a bulky enlargement of the kidney, a condition considered as a premalignant state before WT. Despite relevant clinical differences between WT and DHPLN, they are often challenging to distinguish based on histology. Molecular markers would improve differential diagnosis, but none are available at present. In our study, we investigated the potential of microRNAs (miRNAs) as such biomarkers, also aiming to shed light on the chronological order of expression changes. Formalin-fixed, paraffin-embedded (FFPE) samples from four DHPLN cases and adjacent healthy tissues were tested using a PCR array containing primers for 84 miRNAs implicated in genitourinary cancer. Expression in DHPLN was compared to WT data available in dbDEMC. Let-7, miR-135, miR-146a-5p, miR-182-5p, miR-183-5p, miR-20b-3p, miR-29b-3p, miR-195-5p and miR-17-5p showed potential to be used as biomarkers to distinguish WT and DHPLN in cases when traditional differential diagnosis is inconclusive. Our study also revealed miRNAs which may play a role in the initial steps of the pathogenesis (at a precancerous stage) and ones which become deregulated later in WT. More experiments are needed to confirm our observations and find new candidate markers.

## 1. Introduction

Wilms’ tumor (WT) is the most common malignant renal tumor in children [1] and is generally diagnosed before the age of 5 [2]. Although most cases are sporadic, some Wilms’ tumors display genetic predisposition [3,4,5]. In Europe, treatment involves 4–6 weeks of preoperative chemotherapy as recommended by the International Society of Paediatric Oncology (SIOP), while in the United States, Children’s Oncology Group’s protocols are applied, with nephrectomy as a first step [6].

**Figure 1:**
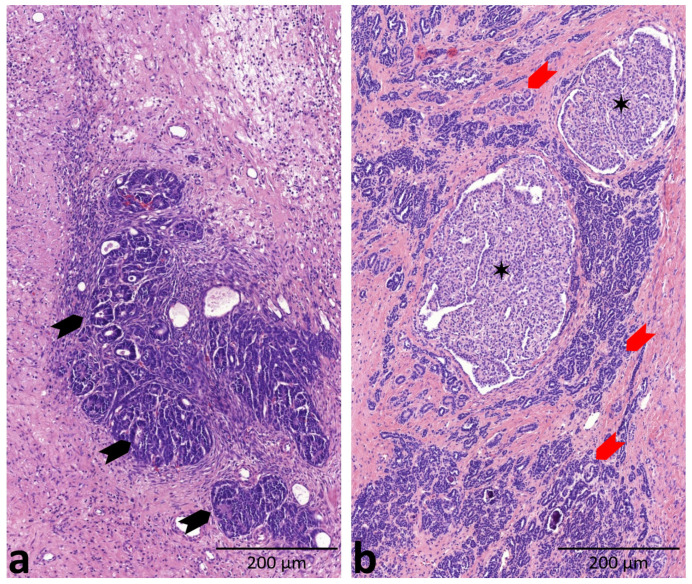
Morphological features of a Wilms’ tumor (WT) and a nephrogenic rest (NR). (**a**) Regressive WT is composed of primitive tubules (black arrows) resembling the developing kidney parenchyma. (**b**) An NR built up by atrophic, primitive-like tubular structures (red arrows). Two hyperplastic areas are also present (black asterisks). Source: own images (magnification: 200×).

Nephrogenic rests (NRs) are remnants found when embryonic elements persist beyond 36 weeks of intrauterine development [7]. These rests are classified based on their location and histology, and multifocal or diffuse rests have the highest clinical relevance. Perilobar NRs are located at the periphery of the renal lobe, while intralobar NRs are found within the lobe. Most NRs stay in a dormant phase or regress, while some transform into a hyperplastic form or even WT [8]. Diffuse hyperplastic perilobar nephroblastomatosis (DHPLN) is a condition in which perilobar NRs are so abundant that they result in a bulky enlargement of the kidney, considered as a precancerous lesion, especially for bilateral cases of WT [9]. Differential diagnosis of WT and DHPLN is traditionally based on radiological and histological features [10] and the presence or absence of a fibrous pseudocapsule. In Europe, however, a fibrous capsule similar to the one seen in WT may develop in DHPLN as a result of preoperative chemotherapy applied as part of the SIOP protocol, while WT cases of the blastemal type may lack any form of capsule [7]. In medical practice, a small WT nodule may appear nearly or fully indistinguishable from a NR seen in DHPLN (Figure 1), despite the relevant difference (malignant or premalignant) between their pathology and treatment requirements. This calls for molecular markers to reliably identify each disorder [11].

However, such markers have never been reported until now. Some authors suggest that the genetic landscape of DHPLN may look somewhat similar to bilateral/syndromic WT, including a loss of the Wilms’ tumor 1 (*WT1*) gene [12,13], while others point to mutations in genes such as SIX Homeobox 1 (*SIX1*), Drosha ribonuclease III (*DROSHA*) and DiGeorge Critical Region 8 (*DGCR8*), which are (rarely) seen in high-risk blastemal WT and have never been observed in nephroblastomatosis [14]. Methylation patterns are also similar in NRs and WT, but blastemal elements tend to display an overexpression of insulin-like growth factor 2 (IGF2), considered as an early driver of malignant transformation. This is often caused by a loss of imprinting at 11p15 [15]. While not all (syndromic or non-syndromic) WTs develop from a premalignant lesion such as DHPLN, genetic syndromes that typically feature WT also predispose to nephroblastomatosis: WAGR (WT, aniridia, genitourinary malformations and mental retardation) and Denys–Drash syndrome are characterized by a deletion or an exonic mutation affecting the *WT1* gene, respectively, while Beckwith–Wiedemann (methylation changes at 11p15) and Perlman syndrome do not involve *WT1* but still increase predisposition [11]. In summary, the currently available evidence is insufficient to differentiate between DHPLN and WT on a genetic basis.

MicroRNAs (miRNAs) are small, endogenous non-coding RNAs with a length of 18–22 nucleotides. Their regulatory ability is mostly exerted at the post-transcriptional level through a cleavage or translational repression of target mRNAs. miRNAs play a role in many cancer types and are classified as oncogenes or tumor suppressors [16]. In the present study, for the first time, to our knowledge, we aimed to assess whether miRNAs may be used as biomarkers to differentiate between DHPLN and WT. In cooperation with three Hungarian pathology institutes, we analyzed formalin-fixed, paraffin-embedded (FFPE) samples of four DHPLN patients, comparing miRNA expression levels with WT. Our results may also shed more light on the chronological order of steps in WT pathogenesis, i.e., which miRNAs are deregulated even at a precancerous stage and which changes occur later (in WT).

Our low sample size limited the application of inferential statistics, and as a result, interpretation of our data is mostly qualitative and not quantitative. While obtaining a high number of DHPLN samples (also containing healthy regions) could be challenging with any study design, we do encourage further research as an established miRNA profile distinguishing DHPLN from WT may considerably improve patient care.

## 2. Results

Two out of the eighty-four miRNAs we studied, hsa-miR-3662 and hsa-miR-3666, were excluded due to a lack of amplification in any of the samples. Most other miRNAs were successfully amplified in all four samples (Table S1). Means of log_2_ fold change (log_2_FC) values were calculated to evaluate expression patterns characteristic to DHPLN. While DHPLN results were obtained only from our FFPE samples, WT results from kidney tissue (FFPE and fresh) and blood samples were available in the literature [14,17,18,19,20,21] alongside our single FFPE sample from Patient 1 (we included results from all relevant studies featured in the dbDEMC database). These are shown in Table S2. In Table 1, mean fold changes of each miRNA are outlined for DHPLN and WT. Apart from mean values, scatters and literature reports were assessed for each miRNA, and findings thought to be the most relevant are discussed in Section 3.2.

In the case of five miRNAs (miR-128-3p, miR-34c-5p, miR-184, miR-194-5p and miR-203a-3p), Student’s *t*-tests were applied to FFPE-based data (see Section 4.6). For miR-128-3p, miR-184, miR-194-5p and miR-203a-3p, *p* values ranged between 0.63 and 0.82 (as shown in Table S3). This confirmed observations that these miRNAs are likely to be expressed at about the same level in DHPLN and WT samples. For miR-34c-5p, we obtained *p* = 0.21, which implies the possibility of an expression difference (difficult to assess due to low sample sizes and high scatter).

**Figure 2:**
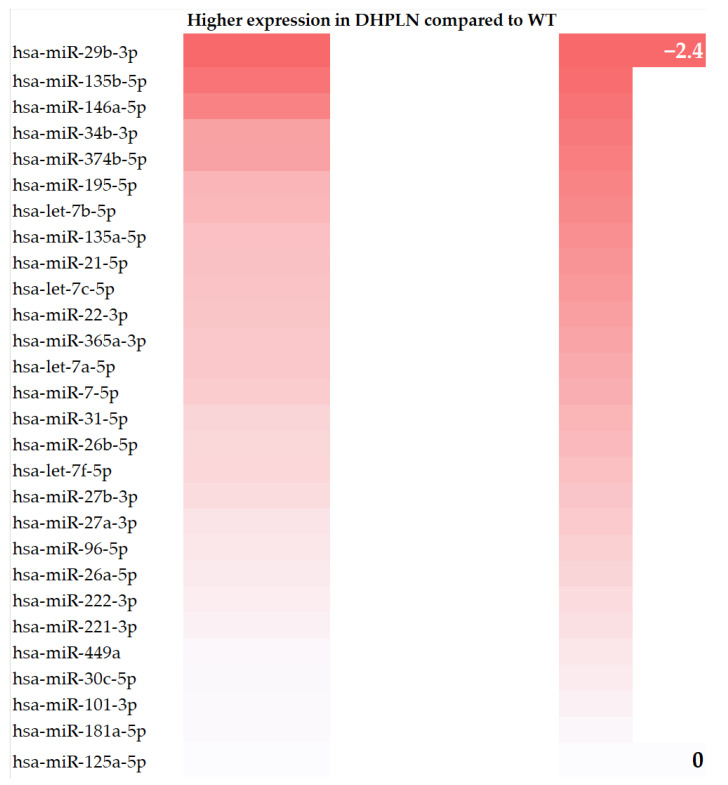

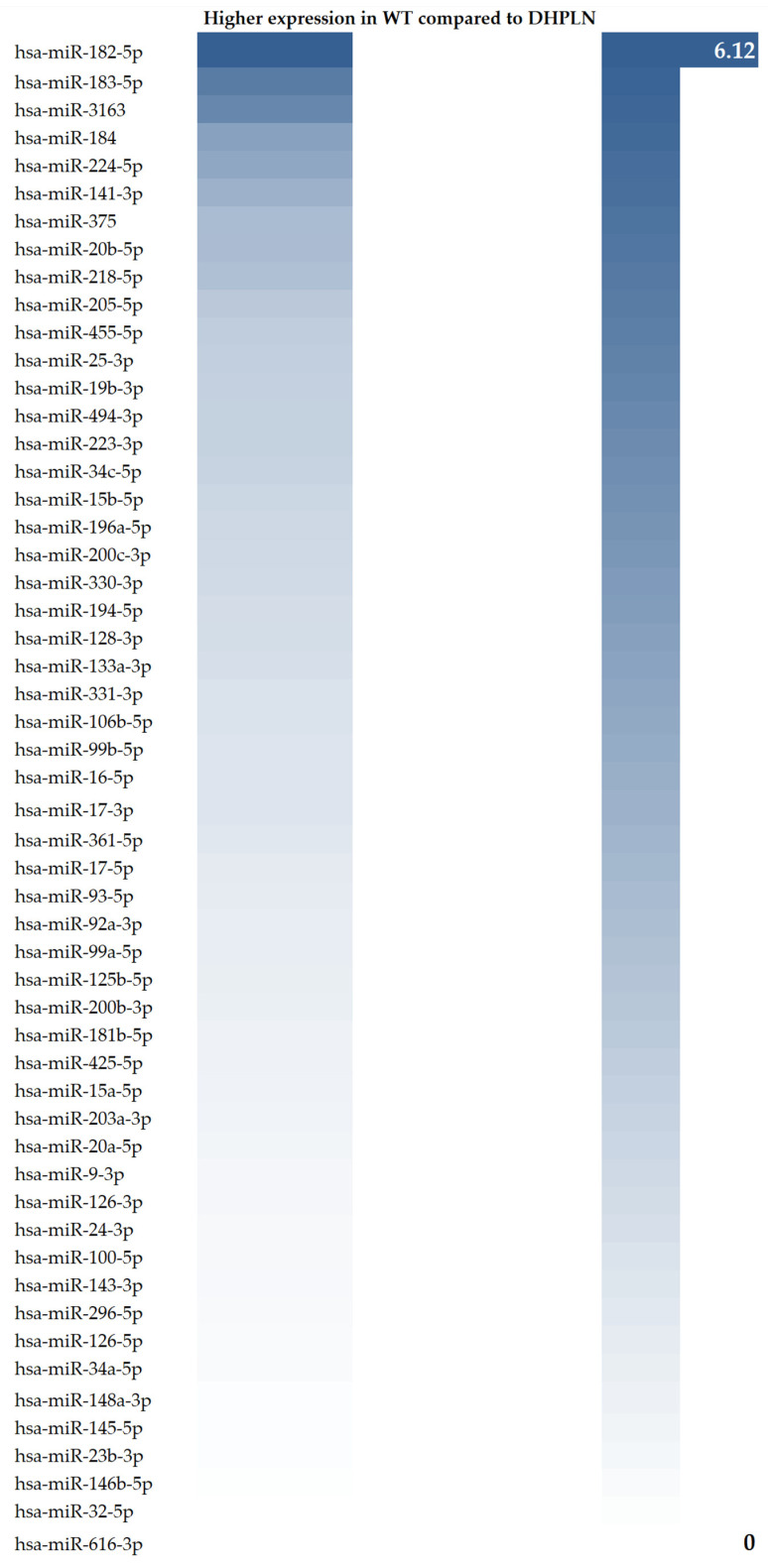
Classification of miRNAs based on expression differences between DHPLN and WT.

**Figure 3:**
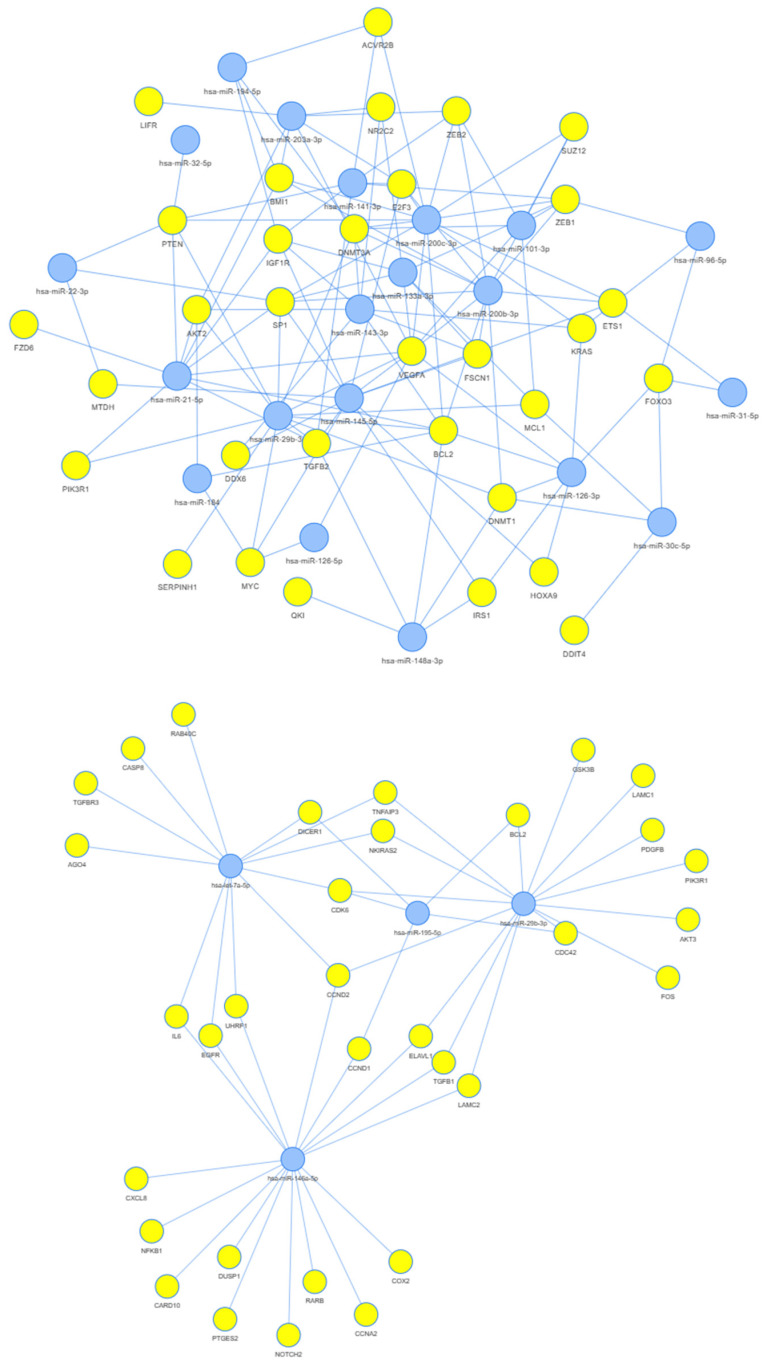

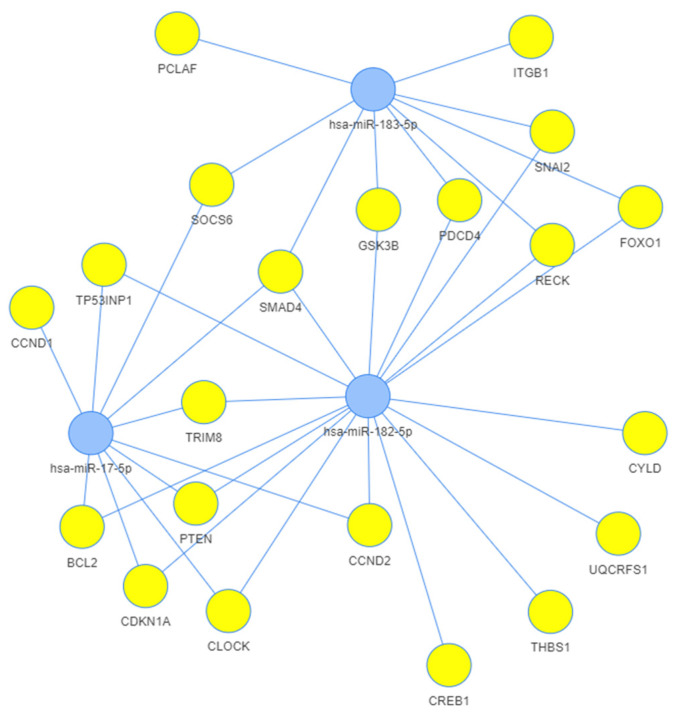
Network analysis of targets for miRNAs that seem to function as tumor suppressors starting from the premalignant stage (**above**), tumor suppressor miRNAs lost during the malignization process (**center**) and oncogenic miRNAs that seem to become elevated as part of the malignization process (**below**).

A number of miRNAs showed considerably different expressions between DHPLN and WT, even if these findings could not be supported by statistical methods given the low number of samples (Figure 2). In Figure 3, we present the results of our network analyses. A large number of tumor suppressor miRNAs were found to be underexpressed even at the premalignant stage DHPLN. Several common targets were also identified for tumor suppressor and oncogenic miRNAs that seem to become deregulated later, as DHPLN progresses into WT (Figure 3). A functional enrichment analysis of miRNAs expressed differentially between DHPLN and WT revealed the following common terms relating to cellular and molecular pathways: cell death, cell cycle, aging, apoptosis, cell proliferation, hematopoiesis, cell differentiation, epithelial-to-mesenchymal transition, hormone-mediated signaling pathway, inflammation and innate immunity.

## 3. Discussion

### 3.1. Clinical Relevance

Perilobar nephrogenic rests are present in about 0.5 percent of the general population, but only a small fraction of these are classified as DHPLN [22]. Without treatment, DHPLN has a high (but not exactly known) likelihood to progress into Wilms’ tumor, while conservative management with prolonged chemotherapy may successfully prevent the development of WT in about half of the cases [23]. Essentially, a well-grounded differential diagnosis between DHPLN and WT would allow much better patient care. While miRNA profiling seems promising in that aspect, such efforts have been scarce.

**Figure 4:**
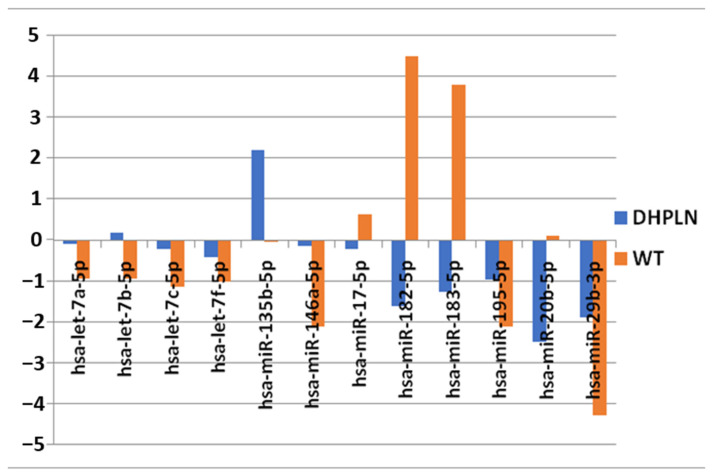
Expression differences of selected miRNAs (in log_2_FC) between DHPLN and WT (see main text).

Senanayake et al. compared the expression of a narrow set of five miRNAs between WT tissue and incidental NRs from the same surgical specimens [24]. Gadd et al. performed genomic and expression analyses of 5 DHPLN cases comparing them to WT, and briefly commented on found miRNA expression differences in a conference paper [25], which was not followed by a full-length publication. These seem to be the only reports available in the literature on the topic and neither one provides numerical expression data. Our present paper addresses the gap in current knowledge by offering insight into the differences of miRNA expression patterns between DHPLN and WT. In the case of several miRNAs, promising results were obtained (Figure 4).

### 3.2. Results in Literature Context

As most data in this paper could not be assessed by inferential statistical methods as explained in Section 4.6, we hereby present our own (mostly qualitative) interpretation.

The following tumor suppressor miRNAs seem to be underexpressed in both DHPLN and WT: miR-101-3p, miR-126-3p, miR-126-5p, miR-133a-3p, miR-141-3p, miR-143-3p, miR-145-5p, miR-148a-3p, miR-184, miR-194-5p, miR-200b-3p, miR-200c-3p, miR-203a-3p, miR-21-5p, miR-22-3p, miR-29b-3p, miR-30c-5p, miR-31-5p, miR-32-5p, miR-455-5p, miR-9-3p and miR-96-5p. We propose that their deregulation probably occurs during the early steps of WT pathogenesis, resulting in detectable changes even at a precancerous stage. Among these tumor suppressors, miR-141, miR-194 and miR-200c were shown before to be underexpressed in both WTs and adjacent nonmalignant NRs, although the amplitudes of decrease were somewhat different from what we observed in DHPLN [24].

Among miRNAs that were differentially expressed, all studied members of the Let-7 family of miRNAs (Let-7a-5p, Let-7b-5p, Let-7c-5p, Let-7f-5p) were expressed higher in DHPLN than in WT. This result correlates with the only available previous report [25], but low sample sizes should be kept in mind to avoid overinterpretation (four in the present study and five in the earlier one). Among the four miRNAs, the largest difference in log_2_FC value was obtained with Let-7b-5p. LIN28 is known to be overexpressed in WT, downregulating the tumor suppressor Let-7 miRNAs in control of the Ras pathway [26]. LIN28 overexpression has been suggested to be directly involved in WT tumorigenesis based on earlier experiments on mouse models [27]. Our results, when viewed together with the report by Gadd et al., suggest that Let-7 deregulation may indeed be directly associated with the malignization process, not present in precancerous DHPLN [25]. This needs to be confirmed in the future.

**Figure 5:**
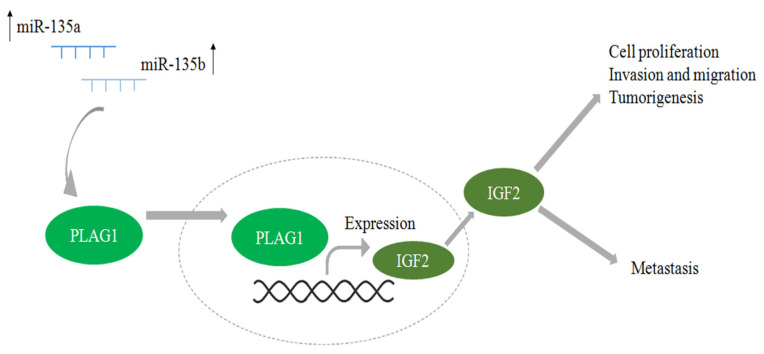
Higher expression of miR-135 variants in DHPLN may prevent malignization by the repression of transcription factor pleomorphic adenoma gene 1 (PLAG1), which plays a role in cell proliferation, invasion, migration and tumorigenesis via a number of targets including insulin-like growth factor 2 (IGF2). When these miRNAs are lost, PLAG1 may become overexpressed and promote the formation of WT [25,28]. Source: own image.

Both miR-135 variants (a and b) were expressed higher in DHPLN (in two out of our four samples) than in WT, where the expression is hardly different from control tissue. Some literature data suggest that an upregulation of miR-135 may cause a repression of the transcription factor pleomorphic adenoma gene 1 (PLAG1) with PLAG1 upregulated in most WTs but downregulated in DHPLN [25]. PLAG1 plays a role in cell proliferation through the regulation of many target genes such as growth factors (e.g., IGF2) [28]. A clonal-increased expression of PLAG1 within DHPLN (possibly due to a change in miR-135 expression at some point) may be suggested as a possible factor in WT tumorigenesis [25], with our own results in support of this view (Figure 5).

miR-146a-5p did not show anything other than a minimal change in any of our four DHPLN samples (mean log_2_FC = −0.13), while it is a known tumor suppressor in WT [18], showing a downregulation in both fresh and FFPE samples (mean log_2_FC = −2.11). This particular miRNA, while somewhat understudied in WT and never reported before in DHPLN, seems to be a strong candidate for a potential miRNA panel aiming to distinguish WT expression signatures from DHPLN. According to our findings, its expression may decrease during the malignization process. miR-146a-5p has been specifically described as a tumor suppressor responsible for metastasis in clear cell renal cell carcinoma, but it functions as an oncogene in some other malignancies [29]. A related miRNA, miR-146b-5p, which differs in only two nucleotides and shares many functions [30], has been shown to be under a bidirectional control by the two major isoforms of the most well-known Wilms’ tumor suppressor, WT1, and is slightly elevated in WT overall [31]. Interestingly, miR-146b-5p expressions we detected (while variable among DHPLN samples) were not that different from miR-146a-5p on average (Table 1). It seems possible that the striking differences between miR-146a and -146b expressions reported in the literature of WT are characteristic to the malignant stage only.

miR-17-5p is a known oncogenic miRNA that tends to be slightly elevated in WT tissue but not in peripheral blood (Table S2), while in our DHPLN cases, it was underexpressed in three out of four samples and elevated only in one (Table S1). In contrast, miR-181 family oncogenes seem to be about equally overexpressed in DHPLN and WT, suggesting a deregulating event early in the pathogenesis (Table 1).

miR-182-5p showed the largest difference (6.12) between the log_2_FC values seen in DHPLN and WT samples. In WT, this miRNA may function as an oncogene, overexpressed both in fresh kidney tissue and in FFPE samples, still unstudied in blood samples to our knowledge (Table S2). There was a high variation of expression levels for this miRNA among the four DHPLN samples, with two of them showing marked underexpression and one displaying an elevation characteristic to WT. Interestingly, the latter case was Patient 1 who went on to develop WT two years later. There are several possible explanations for the observed variability, including the heterogeneity of the molecular profile or the occurrence of an expression switch at some point during the lesion’s progression. miR-182-5p may play a role in the transformation of DHPLN to WT, but further studies are needed to confirm this. In some other tumors of notably different pathogenesis, miR-182-5p suppresses the migration, invasion and colony formation of cancer cells when its expression is increased. This has been reported in colon cancer [32] and bladder cancer [33]. It inhibits the proliferation of renal cell carcinoma (RCC) through the activation of the AKT/FOXO3a signaling pathway [34].

Obtained miR-183-5p expression values were strikingly similar to miR-182-5p in each DHPLN sample. miR-183-5p typically shows elevated expression in WT, somewhat less so in blood. Some authors have suggested that miR-182-5p and miR-183-5p may occasionally act on the same pathways, e.g., in Parkinson’s disease and mesothelioma [35,36], but there are no such reports for WT, possibly due to a lack of data.

miR-184 seems underexpressed in both DHPLN and WT, more prominently in the former. Literature data from blood tests seem questionable, showing a slight increase in sharp contrast with fresh or FFPE kidney tissue samples (Table S2). miR-184 may act as a general tumor suppressor in genitourinary neoplasms. Apart from WT, it plays an important role in RCC as well, where it is also downregulated [37].

We found that the expression of miR-195-5p was not or only slightly decreased in most DHPLN samples, while in WT the expression loss is usually more pronounced. According to the CSmiRTar database, a validated target of miR-195-5p is PLAG1. This may contribute to PLAG1 expression being lower in DHPLN than in WT, an observation made earlier by Gadd et al. [25].

Both arms of the precursor mir-20b, miR-20b-5p and miR-20b-3p, are involved in the pathogenesis of various malignant and non-malignant conditions, with both tumor suppressor and oncogenic roles reported in the literature [38]. In our study, interesting log_2_FC values were obtained for miR-20b-5p. A prominent decrease in expression in all nephroblastomatosis samples (mean log_2_FC = −2.48) may advocate this miRNA as another candidate for a biomarker panel meant to distinguish premalignant and malignant lesions, as a minimal change or slight increase is typical in WT (mean log_2_FC = 0.11). However, peripheral blood is not likely to be suitable for the differential diagnosis as a subset of WT patient samples show a reduced expression [19]. Notably, WT1 may downregulate miR-20b-5p based on evidence from cell lines [31], so WT1-mutant subsets of DHPLN and WT may display higher levels of expression.

miRNAs belonging to the miR-29 family are associated with various cancer pathways, with 677 human genes identified as potential targets according to the miRNet database. In many tumors, these miRNAs display a low expression, affecting the development of metastasis and overall prognosis [39]. In WT, miR-29b-3p regulates a number of Polycomb proteins and may be induced or repressed by WT1 [31]. It is also known to be massively downregulated in WT tissue but not in the blood of WT patients (Table S2). Our results show that miR-29b-3p is underexpressed in DHPLN as well, but less so than in WT. Similarly to miR-20b-5p, it may turn out to be useful for the differential diagnosis, provided that tumor tissue is used as the miRNA source.

With a number of miRNAs, including miR-205-5p, miR-218-5p, miR-224-5p, miR-25-3p, miR-3163, miR-34c-5p and miR-375, we may not exclude the possibility of relevant expression differences, but any interpretation of the data would be shaky at this point, due to either a considerable scatter within our already small sample pool or an absence of PCR amplification in one or more DHPLN samples (Table S1).

### 3.3. Network Analysis

A number of oncogenes playing a role in WT development may be deregulated even at the precancerous stage, based on our observation that many of the associated miRNAs are already lost in DHPLN (Figure 3, top). These include specificity protein 1 (SP1), vascular endothelial growth factor A (VEGFA) and B-cell lymphoma and leukemia antigen 2 (BCL2). SP1 is highly expressed in uninduced kidney mesenchyme and immature podocytes, and it binds to an enhancer of *WT1* regulating its expression [40]. VEGFA is an angiogenic growth factor that is essential in kidney development and also plays a role in the increased microvascularization of WT [41]. BCL2 is an oncoprotein inhibiting apoptosis under control by WT1. Intense BCL2 staining was reported in the blastemal and epithelial elements of both NRs and WTs [42]. Our current results support and may explain these earlier findings.

In WT, cyclin D2 (CCND2) and its partner, cyclin-dependent kinase 6 (CDK6), are each under control by three miRNAs that seem to become underexpressed only in WT (Figure 3, center). According to some preliminary results by Xu et al. (not peer-reviewed yet), CCND2 overexpression may represent a common downstream malignization pathway upon which various types of WT mutation pathways converge [43].

Looking at miRNAs elevated in WT but not in DHPLN according to our findings (Figure 3, bottom), all three of them share a single target: SMAD family member 4 (SMAD4). SMAD4 has long been known to affect the expression of WT1 in cultured podocytes [44] and, more recently, the TGF-β/SMAD pathway was suggested to play a role in WT proliferation and invasion [45].

## 4. Materials and Methods

### 4.1. Patients

FFPE samples were obtained from DHPLN and adjacent healthy kidney tissue from each patient in cooperation with the departments of pathology of three Hungarian universities: Semmelweis University, Universities of Szeged and Pécs (Table 2). Only a single tissue block was preserved per patient, so intralesional variations (and interlesional ones within the same patient in the case of bilateral disease) could not be studied. In Patient 1, WT tissue (taken two years after the nephroblastomatosis) was also available, alongside DHPLN and normal tissue, for comparison of miRNA expression levels (Table S4).

In the case of Patient 4, comorbidities (esophageal atresia, hypospadias, patent foramen ovale, persistent ductus arteriosus and torticollis) were present. The case might represent a syndromic form of the disease as DHPLN and hypospadias may be considered suggestive for WT1 involvement, while various cardiovascular anomalies have been reported in Beckwith–Wiedemann syndrome, which may occasionally manifest without cardinal features such as macroglossia and hemihypertrophy [46]. Unfortunately, this would be challenging to investigate from an FFPE sample, and the patient is out of our reach.

### 4.2. Sample Handling

FFPE samples were prepared according to the UMBRELLA protocol. After surgical removal, samples were fixed in formaldehyde (24 h), followed by a dehydration protocol that involved gradually increasing concentrations of ethanol. Finally, samples were embedded in paraffin wax.

### 4.3. RNA Extraction and Reverse Transcription

We followed laboratory protocols as published before by us and others [20,21,47]. For miRNA extraction, miRNeasy FFPE Kits by Qiagen (Hilden, Germany) were used. Reverse transcription was performed using miRCURY LNA RT Kits (Qiagen). The concentration of RNA in each sample was assessed by NanoDrop. Amounts of miRNA extracted from FFPE samples are generally much lower than expected on the basis of concentration measurements due to the presence of degraded RNA fragments in the size range of miRNA [48]. Accordingly, the reverse transcription protocol provided by the manufacturer was slightly modified to include 200 ng (instead of 20 ng) RNA template per reaction mix. Total reaction volumes for each PCR assay were 20 µL. After reverse transcription, cDNA was stored at −20 °C until a PCR experiment.

### 4.4. Studying miRNA Expression Levels with qRT-PCR Arrays

miRCURY LNA miRNA Focus PCR Panels (Qiagen) and a miRCURY LNA SYBR Green PCR Kit (Qiagen) in the qRT-PCR experiments were used. Eighty-four individual forward primers against specific miRNA targets relevant in Wilms’ tumor as well as other types of genitourinary cancer were present on the PCR array (a universal reverse primer was used). In addition, the array contained forward primers against four endogenous controls: Small Nucleolar RNA, C/D Box 44 (SNORD44), Small Nucleolar RNA, C/D Box 38B (SNORD 38B), Small Nucleolar RNA, C/D Box 49A (SNORD49A) and U6 small nuclear RNA (U6 snRNA).

Amplification of control and DHPLN cDNA samples (and the tumor sample from Patient 1) was performed with a Roche LightCycler 96 PCR system (Roche, Basel, Switzerland). According to the manufacturer’s instructions, we used the total reaction volume (20 μL) of cDNA from the reverse transcription. PCR conditions were the following: a 2-min heat activation at 95 °C, then 2-step cycling (denaturation for 10 s at 90 °C, and combined annealing/extension for 60 s at 56 °C) continued for 45 cycles. Amplification was followed by a melting curve analysis (95 °C for 60 s, 40 °C for 60 s then 65 °C to 97 °C with a ramp of 0.07 °C/s). In wells that produced a Ct value over 40 or a dubious melting curve with a Ct between 35 and 40, the result was considered negative (no detectable expression). LightCycler^®^96 software (Roche, version 1.1) and the ∆∆Ct method [49] were used for data evaluation. Control Ct values were defined as the average of the four endogenous controls found on the PCR array.

Fold change (FC) was defined by the formula 2^−∆∆Ct^. FC values are presented in our paper on a logarithmic scale (thus, log_2_FC equals the opposite of ∆∆Ct). According to the above, each original datapoint for each miRNA represents a single measurement of difference between two samples of the FFPE tissue for each patient, one taken from a region of DHPLN, and the other from a region of normal kidney tissue. This measurement reflects the difference (expressed as log_2_FC) between the observed miRNA expression in the DHPLN region compared with the expression in a normal control region of the same tissue block (defining the latter as the log_2_FC baseline for that patient). A mean log_2_FC representing DHPLN was calculated from the four patients for each miRNA and compared to data previously published in WT.

### 4.5. Network Analysis

We performed a network analysis for targets of miRNAs that seemed relevant in our experiments (see below) using the online tool MIENTURNET to tailor results from miRTarBase [50]. Our query was filtered for strong experimental evidence only, with an adjusted *p* value (FDR) of 0.05 and a threshold for the minimum number of miRNA-target interactions set according to the number of found interactions so as not to overpopulate our figures (the threshold was 5 for tumor suppressor miRNAs underexpressed in both WT and DHPLN and 2 for the rest). We also performed a functional enrichment analysis on miRNAs expressed differentially between DHPLN and WT using the TAM 2.0 database [51].

### 4.6. Statistical Analysis

A limited statistical analysis was performed to compare mean expression values detected in DHPLN and WT FFPE samples, the latter group including one WT sample from the current project and ones studied earlier in our lab using the same laboratory procedure [20,21]. miRNAs that were only detected from two or three WT FFPE samples were excluded (Table S2), leaving only five miRNAs, all of which were studied in nine or more WT samples: miR-128-3p, miR-34c-5p, miR-184, miR-194-5p and miR-203a-3p. Student’s *t*-tests for independent samples were carried out using the MedCalc software (version 20.305).

## 5. Conclusions

Our results may contribute to a better understanding of WT pathogenesis by shedding some light on the chronological order of miRNA deregulations reported earlier. By examining the expression of a relatively large set of miRNAs, we may conclude that such markers have the potential to complement traditional diagnoses in cases when differentiating between a small WT nodule or a NR is challenging. Considering our results within the literature context, we propose that the Let-7 and miR-135 family of miRNAs, along with miR-146a-5p, miR-182-5p, miR-183-5p, miR-20b-3p, miR-29b-3p, miR-195-5p and miR-17-5p, are worth studying further to confirm or reject their usefulness in a future biomarker panel. Because of the extreme rarity of newly diagnosed DHPLN cases, utilization of FFPE samples as miRNA sources will likely be decisive on the way towards that goal.

## Figures and Tables

**Table 1:** An overview of miRNA expressions (log_2_FC) in DHPLN and WT. The second column comprises log-differences from expression levels in adjacent normal tissue from the same tissue blocks. The third column mainly contains literature data (see main text and Table S2 for the details on how weighted average values were obtained). The difference between WT and DHPLN is shown in the last column.

miRNA	DHPLN	WT, Weighted Average	WT—DHPLN
hsa-let-7a-5p	−0.08	−0.93	−0.85
hsa-let-7b-5p	0.18	−0.92	−1.10
hsa-let-7c-5p	−0.2	−1.14	−0.94
hsa-let-7f-5p	−0.41	−1.02	−0.61
hsa-miR-100-5p	−0.43	−0.13	0.30
hsa-miR-101-3p	−1.44	−1.50	−0.06
hsa-miR-106b-5p	−0.14	1.02	1.16
hsa-miR-125a-5p	0.02	0.00	−0.02
hsa-miR-125b-5p	−0.69	0.01	0.70
hsa-miR-126-3p	−2.32	−1.95	0.37
hsa-miR-126-5p	−1.96	−1.71	0.25
hsa-miR-128-3p	0.02	1.38	1.36
hsa-miR-133a-3p	−2.56	−1.28	1.28
hsa-miR-135a-5p	1.46	0.49	−0.97
hsa-miR-135b-5p	2.2	0.00	−2.20
hsa-miR-143-3p	−1.83	−1.54	0.29
hsa-miR-141-3p	−5.51	−2.49	3.02
hsa-miR-145-5p	−1.96	−1.85	0.11
hsa-miR-146a-5p	−0.13	−2.11	−1.98
hsa-miR-146b-5p	0.64	0.72	0.08
hsa-miR-148a-3p	−1.17	−1.05	0.12
hsa-miR-15a-5p	−1.2	−0.67	0.53
hsa-miR-15b-5p	−0.26	1.31	1.57
hsa-miR-16-5p	−0.99	0.08	1.07
hsa-miR-17-5p	−0.21	0.63	0.84
hsa-miR-17-3p	−0.36	0.70	1.06
hsa-miR-181a-5p	1.43	1.37	−0.06
hsa-miR-181b-5p	1.62	2.18	0.56
hsa-miR-182-5p	−1.61	4.51	6.12
hsa-miR-183-5p	−1.26	3.80	5.06
hsa-miR-184	−4.74	−1.09	3.65
hsa-miR-194-5p	−5.04	−3.68	1.36
hsa-miR-195-5p	−0.97	−2.12	−1.15
hsa-miR-196a-5p	0.42	1.97	1.55
hsa-miR-19b-3p	−1.05	0.77	1.82
hsa-miR-200b-3p	−4.72	−4.06	0.66
hsa-miR-200c-3p	−4.21	−2.72	1.49
hsa-miR-203a-3p	−4.17	−3.67	0.50
hsa-miR-205-5p	−0.47	1.67	2.14
hsa-miR-20a-5p	−0.26	0.18	0.44
hsa-miR-20b-5p	−2.48	0.11	2.59
hsa-miR-21-5p	−0.97	−1.93	−0.96
hsa-miR-218-5p	−1.25	1.22	2.47
hsa-miR-22-3p	−2.36	−3.26	−0.90
hsa-miR-221-3p	−0.72	−0.91	−0.19
hsa-miR-222-3p	−0.47	−0.73	−0.26
hsa-miR-223-3p	−0.56	1.25	1.81
hsa-miR-224-5p	−1.51	1.89	3.40
hsa-miR-23b-3p	−0.33	−0.22	0.11
hsa-miR-24-3p	−0.59	−0.27	0.32
hsa-miR-25-3p	0.39	2.27	1.88
hsa-miR-26a-5p	−0.39	−0.69	−0.30
hsa-miR-26b-5p	−0.25	−0.86	−0.61
hsa-miR-27a-3p	−0.79	−1.19	−0.40
hsa-miR-27b-3p	−0.69	−1.22	−0.53
hsa-miR-296-5p	0.75	1.02	0.27
hsa-miR-29b-3p	−1.89	−4.28	−2.39
hsa-miR-30c-5p	−1.76	−1.84	−0.08
hsa-miR-31-5p	−1.54	−2.18	−0.64
hsa-miR-3163	−4.16	0.47	4.63
hsa-miR-32-5p	−1.3	−1.27	0.03
hsa-miR-330-3p	−0.6	0.86	1.46
hsa-miR-331-3p	−0.72	0.44	1.16
hsa-miR-34a-5p	0.35	0.57	0.22
hsa-miR-34b-3p	0.1	−1.37	−1.47
hsa-miR-34c-5p	−2.28	−0.55	1.73
hsa-miR-361-5p	−0.3	0.68	0.98
hsa-miR-365a-3p	−0.52	−1.37	−0.85
hsa-miR-374b-5p	0.22	−1.24	−1.46
hsa-miR-375	−2.69	−0.07	2.62
hsa-miR-425-5p	0.07	0.63	0.56
hsa-miR-449a	1.24	1.14	−0.10
hsa-miR-455-5p	−2.71	−0.77	1.94
hsa-miR-494-3p	−0.88	0.94	1.82
hsa-miR-616-3p	1.04	1.06	0.02
hsa-miR-7-5p	0	−0.79	−0.79
hsa-miR-9-3p	−2.12	−1.74	0.38
hsa-miR-92a-3p	0.09	0.83	0.74
hsa-miR-93-5p	0.29	1.08	0.79
hsa-miR-96-5p	−1.25	−1.62	−0.37
hsa-miR-99a-5p	−1.22	−0.50	0.72
hsa-miR-99b-5p	−0.03	1.05	1.08

**Table 2:** DHPLN patients included in the study. Patient 4 presented with additional congenital abnormalities (see main text). * The date of the first operation. Two years later, the patient developed WT (an FFPE sample was kept and is also included).

Patient ID	Age (Years)	Sex	Date of Surgery	Laterality
1	1	female	2007 *	bilateral
2	4	male	1991	bilateral
3	4	male	2014	right
4	1	male	2016	bilateral

## Data Availability

Additional data is available as Supplementary Materials.

## Supplementary Materials

The following supporting information can be downloaded at: https://www.mdpi.com/article/10.3390/ijms24108793/s1.
