# Supplementary material for: Alterations of miRNA Expression in Diffuse Hyperplastic Perilobar Nephroblastomatosis: Mapping the Way to Understanding Wilms’ Tumor Development and Differential Diagnosis
Source: Int J Mol Sci. 2023 May 15;24(10):8793. doi: 10.3390/ijms24108793 (PMC10218489; doi:10.3390/ijms24108793)
Supplement: Supplementary file 1 [file ijms-24-08793-s001.zip › ijms-2379832-supplementary.pdf]

Table S1. Log_2FC values of DHPLN samples from four patients (1-4).

miRNA	Patient 1	Patient 2	Patient 3	Patient 4	Mean
hsa-let-7a-5p	0.21	-0.35	-0.39	0.23	-0.08
hsa-let-7b-5p	0.34	-0.30	0.14	0.56	0.18
hsa-let-7c-5p	0.03	-0.43	-0.26	-0.15	-0.20
hsa-let-7f-5p	0.07	-0.10	-1.36	-0.24	-0.41
hsa-miR-100-5p	-0.87	-0.87	0.25	-0.22	-0.43
hsa-miR-101-3p	-0.13	-0.53	-2.39	-2.72	-1.44
hsa-miR-106b-5p	2.26	0.14	-0.46	-2.49	-0.14
hsa-miR-125a-5p	0.53	-0.77	-0.77	1.09	0.02
hsa-miR-125b-5p	-1.48	-0.87	-0.17	-0.25	-0.69
hsa-miR-126-3p	-1.26	-3.24	-3.17	-1.62	-2.32
hsa-miR-126-5p	-1.75	-3.49	-1.89	-0.69	-1.96
hsa-miR-128-3p	2.31	0.29	-1.41	-1.11	0.02
hsa-miR-133a-3p	-4.50	-1.73	-3.71	-0.30	-2.56
hsa-miR-135a-5p	1.92	0.41	3.09	0.42	1.46
hsa-miR-135b-5p	3.58	-0.49	5.06	0.67	2.20
hsa-miR-141-3p	-3.93	-4.89	-7.53	-5.69	-5.51
hsa-miR-143-3p	-2.49	-0.93	-0.86	-3.03	-1.83
hsa-miR-145-5p	-2.62	-1.79	-1.51	-1.92	-1.96
hsa-miR-146a-5p	-0.46	-0.20	-0.79	0.92	-0.13
hsa-miR-146b-5p	1.86	-0.83	-0.16	1.68	0.64
hsa-miR-148a-3p	-0.14	0.25	-2.24	-2.53	-1.17
hsa-miR-15a-5p	-0.55	-0.40	-1.58	-2.27	-1.20
hsa-miR-15b-5p	1.42	0.45	-1.15	-1.76	-0.26
hsa-miR-16-5p	0.34	-1.23	-2.12	-0.94	-0.99
hsa-miR-17-5p	2.42	-0.55	-1.17	-1.53	-0.21
hsa-miR-17-3p	1.88	-0.38	-1.61	-1.33	-0.36
hsa-miR-181a-5p	1.58	1.12	2.04	0.98	1.43
hsa-miR-181b-5p	1.80	0.86	1.69	2.15	1.62
hsa-miR-182-5p	2.78	-3.64	-6.14	0.56	-1.61
hsa-miR-183-5p	2.08	-2.88	-4.88	0.63	-1.26
hsa-miR-184	-2.71	-4.85	-6.93	-4.47	-4.74
hsa-miR-194-5p	-2.35	-5.07	-7.71	-5.04	-5.04
hsa-miR-195-5p	0.43	0.00	-2.84	-1.46	-0.97
hsa-miR-196a-5p	2.39	-0.69	-1.04	1.03	0.42
hsa-miR-19b-3p	1.70	-0.61	-1.78	-3.51	-1.05
hsa-miR-200b-3p	-3.59	-2.43	-11.52	-1.35	-4.72
hsa-miR-200c-3p	-3.90	-3.69	-7.69	-1.57	-4.21
hsa-miR-203a-3p	-3.90	-4.21	-5.01	-3.56	-4.17
hsa-miR-205-5p	-1.73	-0.71	-1.26	1.83	-0.47
hsa-miR-20a-5p	2.08	-0.56	-1.30	-1.24	-0.26
hsa-miR-20b-5p	-1.06	-1.82	-3.50	-3.53	-2.48
hsa-miR-21-5p	-1.10	-1.49	-2.32	1.02	-0.97
hsa-miR-218-5p	1.45	-2.58	-2.73	-1.14	-1.25
hsa-miR-22-3p	-2.16	-1.96	-2.34	-2.98	-2.36
hsa-miR-221-3p	1.47	-1.61	-1.47	-1.25	-0.72
hsa-miR-222-3p	1.05	-1.45	-0.84	-0.65	-0.47
hsa-miR-223-3p	-1.00	-0.90	-0.72	0.37	-0.56
hsa-miR-224-5p	-0.05	-2.81	-4.14	0.95	-1.51
hsa-miR-23b-3p	1.23	-2.09	-0.86	0.40	-0.33

hsa-miR-24-3p	0.64	-0.90	-1.17	-0.92	-0.59
hsa-miR-25-3p	2.77	-0.58	-1.21	0.57	0.39
hsa-miR-26a-5p	-0.17	-0.79	-0.81	0.20	-0.39
hsa-miR-26b-5p	0.03	-0.29	-0.69	-0.04	-0.25
hsa-miR-27a-3p	0.20	-1.52	-0.94	-0.90	-0.79
hsa-miR-27b-3p	0.62	-1.40	-1.07	-0.90	-0.69
hsa-miR-296-5p	1.37	1.45	0.01	0.19	0.75
hsa-miR-29b-3p	-3.62	-0.66	-0.75	-2.51	-1.89
hsa-miR-30c-5p	-0.81	-2.68	-1.74	-1.80	-1.76
hsa-miR-31-5p	-0.03	-1.76	-1.14	-3.23	-1.54
hsa-miR-3163	-	-	-4.16	-	-4.16
hsa-miR-32-5p	-0.48	-1.12	-1.55	-2.06	-1.30
hsa-miR-330-3p	0.70	-1.37	-0.52	-1.20	-0.60
hsa-miR-331-3p	-0.09	-0.92	-0.16	-1.70	-0.72
hsa-miR-34a-5p	-0.23	0.49	2.70	-1.54	0.35
hsa-miR-34b-3p	0.32	-	-	-0.12	0.10
hsa-miR-34c-5p	-2.17	-	-1.60	-3.07	-2.28
hsa-miR-361-5p	1.15	-1.15	-1.66	0.45	-0.30
hsa-miR-365a-3p	-0.58	-0.79	-0.41	-0.31	-0.52
hsa-miR-3662	-	-	-	-	-
hsa-miR-3666	-	-	-	-	-
hsa-miR-374b-5p	1.12	-0.63	0.17	0.21	0.22
hsa-miR-375	-0.21	-1.79	-10.13	1.37	-2.69
hsa-miR-425-5p	0.51	-	0.00	-0.31	0.07
hsa-miR-449a	1.24	-	-	-	1.24
hsa-miR-455-5p	-2.41	-	-2.44	-3.28	-2.71
hsa-miR-494-3p	0.67	-	-0.64	-2.68	-0.88
hsa-miR-616-3p	-	-	-0.96	3.05	1.04
hsa-miR-7-5p	-0.06	0.12	1.36	-1.41	0.00
hsa-miR-9-3p	-1.50	-2.85	0.12	-4.26	-2.12
hsa-miR-92a-3p	1.43	-0.65	-1.42	1.02	0.09
hsa-miR-93-5p	2.90	-0.44	0.17	-1.45	0.29
hsa-miR-96-5p	1.10	-0.63	-3.67	-1.81	-1.25
hsa-miR-99a-5p	-2.07	-2.05	0.78	-1.52	-1.22
hsa-miR-99b-5p	0.86	-1.81	0.42	0.42	-0.03

Table S2. Log_2FC values from WT samples of various types. Sample sizes are shown in brackets. Includes literature data [14.17–21] and our single WT sample from Patient 1.

miRNA	Fresh kidney tissue	Blood	FFPE	Weighted average
hsa-let-7a-5p	-	-0.95 (59)	-0.51 (3)	-0.93
hsa-let-7b-5p	-	-0.94 (59)	-0.62 (3)	-0.92
hsa-let-7c-5p	-	-1.17 (59)	-0.49 (3)	-1.14
hsa-let-7f-5p	-	-1.04 (59)	-0.68 (3)	-1.02
hsa-miR-100-5p	-	-	-0.13 (3)	-0.13
hsa-miR-101-3p	-1.80 (77)	-1.01 (43)	-0.65 (3)	-1.50
hsa-miR-106b-5p	2.22 (77)	-0.52 (59)	0.53 (3)	1.02
hsa-miR-125a-5p	-	-	0.00 (3)	0.00
hsa-miR-125b-5p	-	-	0.01 (3)	0.01
hsa-miR-126-3p	-1.60 (77)	-2.52 (43)	-2.70 (3)	-1.95
hsa-miR-126-5p	-	-	-1.71 (3)	-1.71
hsa-miR-128-3p	2.17 (57)	0.57 (43)	0.35 (10)	1.38
hsa-miR-133a-3p	-	-1.44 (43)	1.05 (3)	-1.28
hsa-miR-135a-5p	-	0.51 (59)	0.12 (3)	0.49
hsa-miR-135b-5p	-	-0.28 (16)	1.47 (3)	0.00
hsa-miR-141-3p	-3.30 (82)	-0.83 (43)	-4.00 (3)	-2.49
hsa-miR-143-3p	-2.09 (57)	0.46 (16)	-1.70 (3)	-1.54
hsa-miR-145-5p	-1.89 (57)	-	-1.01 (3)	-1.85
hsa-miR-146a-5p	-2.17 (57)	-	-0.92 (3)	-2.11
hsa-miR-146b-5p	-	0.75 (16)	0.59 (3)	0.72
hsa-miR-148a-3p	-1.66 (57)	1.12 (16)	-0.91 (3)	-1.05
hsa-miR-15a-5p	-0.68 (57)	-	-0.49 (3)	-0.67
hsa-miR-15b-5p	1.36 (57)	1.45 (16)	-0.40 (3)	1.31
hsa-miR-16-5p	-	-	0.08 (3)	0.08
hsa-miR-17-5p	1.67 (77)	-0.73 (59)	0.47 (3)	0.63
hsa-miR-17-3p	1.40 (77)	-0.23 (59)	0.98 (3)	0.70
hsa-miR-181a-5p	1.38 (77)	-	1.17 (3)	1.37
hsa-miR-181b-5p	2.22 (77)	-	1.27 (3)	2.18
hsa-miR-182-5p	4.62 (57)	-	2.32 (3)	4.51
hsa-miR-183-5p	4.82 (57)	0.58 (16)	1.68 (3)	3.80
hsa-miR-184	-2.08 (62)	1.15 (59)	-5.24 (17)	-1.09
hsa-miR-194-5p	-5.37 (139)	0.53 (59)	-4.45 (17)	-3.68
hsa-miR-195-5p	-2.21 (57)	-	-0.40 (3)	-2.12
hsa-miR-196a-5p	-	-	1.97 (3)	1.97
hsa-miR-19b-3p	1.19 (20)	0.62 (43)	0.22 (3)	0.77
hsa-miR-200b-3p	-4.07 (119)	-	-3.73 (3)	-4.06
hsa-miR-200c-3p	-3.11 (119)	0.38 (16)	-3.56 (3)	-2.72
hsa-miR-203a-3p	-	-	-3.67 (17)	-3.67
hsa-miR-205-5p	-	-	1.67 (2)	1.67
hsa-miR-20a-5p	1.42 (77)	-1.45 (59)	0.18 (3)	0.18
hsa-miR-20b-5p	1.48 (57)	-1.18 (59)	-0.42 (3)	0.11
hsa-miR-21-5p	-1.98 (57)	-	-0.91 (3)	-1.93
hsa-miR-218-5p	1.25 (57)	-	0.56 (3)	1.22
hsa-miR-22-3p	-3.31 (77)	-	-2.13 (3)	-3.26
hsa-miR-221-3p	-	-0.95 (43)	-0.40 (3)	-0.91
hsa-miR-222-3p	-	-0.76 (43)	-0.25 (3)	-0.73

hsa-miR-223-3p	-	1.56 (16)	-0.38 (3)	1.25
hsa-miR-224-5p	1.58 (57)	2.42 (43)	0.28 (3)	1.89
hsa-miR-23b-3p	-0.84 (57)	0.59 (43)	0.02 (3)	-0.22
hsa-miR-24-3p	-	-	-0.27 (3)	-0.27
hsa-miR-25-3p	2.45 (77)	1.51 (16)	1.70 (3)	2.27
hsa-miR-26a-5p	-1.02 (57)	-	5.66 (3)	-0.69
hsa-miR-26b-5p	-0.87 (57)	-0.87 (59)	-0.60 (3)	-0.86
hsa-miR-27a-3p	-1.55 (57)	-0.75 (43)	-0.58 (3)	-1.19
hsa-miR-27b-3p	-1.27 (57)	-	-0.35 (3)	-1.22
hsa-miR-296-5p	-	1.00 (43)	1.30 (3)	1.02
hsa-miR-29b-3p	-5.31 (77)	0.51 (16)	-3.51 (3)	-4.28
hsa-miR-30c-5p	-2.72 (139)	1.00 (43)	-1.65 (3)	-1.84
hsa-miR-31-5p	-2.23 (57)	-	-1.18 (3)	-2.18
hsa-miR-3163	-	-	0.47 (2)	0.47
hsa-miR-32-5p	-	-1.33 (43)	-0.34 (3)	-1.27
hsa-miR-330-3p	1.01 (57)	0.40 (16)	0.50 (3)	0.86
hsa-miR-331-3p	0.50 (57)	-	-0.74 (3)	0.44
hsa-miR-34a-5p	-	0.48 (16)	1.03 (3)	0.57
hsa-miR-34b-3p	-	-	-1.37 (3)	-1.37
hsa-miR-34c-5p	-	-0.94 (43)	1.29 (9)	-0.55
hsa-miR-361-5p	0.59 (57)	0.76 (59)	0.67 (3)	0.68
hsa-miR-365a-3p	-1.39 (57)	-	-0.94 (3)	-1.37
hsa-miR-374b-5p	-	-1.33 (43)	0.06 (3)	-1.24
hsa-miR-375	-	-	-0.07 (3)	-0.07
hsa-miR-425-5p	0.66 (57)	-	0.09 (3)	0.63
hsa-miR-449a	-	1.16 (43)	0.75 (2)	1.14
hsa-miR-455-5p	-2.01 (57)	0.91 (43)	-1.34 (3)	-0.77
hsa-miR-494-3p	-	1.01 (59)	-0.54 (3)	0.94
hsa-miR-616-3p	-	-	1.06 (3)	1.06
hsa-miR-7-5p	-	-	-0.79 (3)	-0.79
hsa-miR-9-3p	-	-	-1.74 (3)	-1.74
hsa-miR-92a-3p	1.68 (77)	-0.66 (43)	0.56 (3)	0.83
hsa-miR-93-5p	2.52 (77)	-0.76 (59)	0.33 (2)	1.08
hsa-miR-96-5p	-	-1.71 (43)	0.37 (2)	-1.62
hsa-miR-99a-5p	-	-	-0.50 (3)	-0.50
hsa-miR-99b-5p	1.47 (57)	0.68 (59)	0.30 (3)	1.05

Table S3. A comparison of log_2FC expression values in DHPLN and WT FFPE samples (samples of other types are not included). miRNAs with WT sample sizes of 3 or lower (shown in Table S2) were excluded. To obtain *p* values. t-tests for independent samples were performed.

miRNA	DHPLN mean (sample size)	WT mean (sample size)	WT - DHPLN	*p* value
hsa-miR-128-3p	0.02 (4)	0.35 (10)	0.33	0.8157
hsa-miR-184	-4.74 (4)	-5.24 (17)	-0.5	0.7719
hsa-miR-194-5p	-5.04 (4)	-4.45 (17)	0.59	0.637
hsa-miR-203a-3p	-4.17 (4)	-3.67 (17)	0.5	0.7117
hsa-miR-34c-5p	-2.28 (3)	1.29 (9)	3.57	0.2105

Table S4. Log_2FC values from a single WT sample (Patient 1). also included as one of the FFPE samples in Table S2.

miRNA	WT-log_2FC
hsa-let-7a-5p	0.01
hsa-let-7b-5p	0.25
hsa-let-7c-5p	-0.6
hsa-let-7f-5p	-0.4
hsa-miR-100-5p	0.34
hsa-miR-101-3p	0.02
hsa-miR-106b-5p	1.21
hsa-miR-125a-5p	1.05
hsa-miR-125b-5p	-0.37
hsa-miR-126-3p	-0.97
hsa-miR-126-5p	-1.4
hsa-miR-128-3p	0.2
hsa-miR-133a-3p	-3.19
hsa-miR-135a-5p	1.41
hsa-miR-135b-5p	3.24
hsa-miR-141-3p	-4.35
hsa-miR-143-3p	-3.16
hsa-miR-145-5p	-1.05
hsa-miR-146a-5p	-0.43
hsa-miR-146b-5p	1.92
hsa-miR-148a-3p	-2.81
hsa-miR-15a-5p	-0.81
hsa-miR-15b-5p	-1.85
hsa-miR-16-5p	1.27
hsa-miR-17-5p	1.09
hsa-miR-17-3p	1.48
hsa-miR-181a-5p	1.23
hsa-miR-181b-5p	1.86
hsa-miR-182-5p	2.81
hsa-miR-183-5p	2.06
hsa-miR-184	0.42
hsa-miR-194-5p	-1.43
hsa-miR-195-5p	0.57
hsa-miR-196a-5p	3
hsa-miR-19b-3p	0.68
hsa-miR-200b-3p	-3.21
hsa-miR-200c-3p	-3.66
hsa-miR-203a-3p	-4.79
hsa-miR-205-5p	N/A
hsa-miR-20a-5p	1.18
hsa-miR-20b-5p	-0.07
hsa-miR-21-5p	-0.99
hsa-miR-218-5p	0.74
hsa-miR-22-3p	-2.69
hsa-miR-221-3p	0.79
hsa-miR-222-3p	1.45

hsa-miR-223-3p	0.25
hsa-miR-224-5p	-0.24
hsa-miR-23b-3p	1.56
hsa-miR-24-3p	0.35
hsa-miR-25-3p	3.62
hsa-miR-26a-5p	-0.02
hsa-miR-26b-5p	-0.4
hsa-miR-27a-3p	0.31
hsa-miR-27b-3p	1.13
hsa-miR-296-5p	-0.1
hsa-miR-29b-3p	-4.12
hsa-miR-30c-5p	0
hsa-miR-31-5p	-0.07
hsa-miR-3163	N/A
hsa-miR-32-5p	0.76
hsa-miR-330-3p	1.38
hsa-miR-331-3p	-1.86
hsa-miR-34a-5p	0.78
hsa-miR-34b-3p	-0.81
hsa-miR-34c-5p	N/A
hsa-miR-361-5p	1.64
hsa-miR-365a-3p	-1.69
hsa-miR-3662	N/A
hsa-miR-3666	N/A
hsa-miR-374b-5p	1.59
hsa-miR-375	1.22
hsa-miR-425-5p	0.49
hsa-miR-449a	N/A
hsa-miR-455-5p	-1.64
hsa-miR-494-3p	-1.59
hsa-miR-616-3p	1.65
hsa-miR-7-5p	-1.82
hsa-miR-9-3p	-1.26
hsa-miR-92a-3p	1.36
hsa-miR-93-5p	2.21
hsa-miR-96-5p	0.71
hsa-miR-99a-5p	-1.12
hsa-miR-99b-5p	1.78
